# Distribution and Phytoavailability of Potentially Toxic Metals in Different Fe/Mg Mine Tailings

**DOI:** 10.3390/ijerph15112475

**Published:** 2018-11-06

**Authors:** Xuyin Yuan, Yimin Wang, Doudou Tang, Xiaohui Zhang, Lei Zhang, Haiyan Zhang

**Affiliations:** 1 Key Laboratory of Integrated Regulation and Resources Development of Shallow Lakes of Ministry of Education, Nanjing 210098, China; wangym@hhu.edu.cn (Y.W.); zhangxh_hhu@126.com (X.Z.); lizaoyutian@126.com (L.Z.); hhu_zhy@163.com (H.Z.); 2 College of Environment, Hohai University, Nanjing 210098, China; doubled_tang@163.com

**Keywords:** potential toxic metal, Fe/Mg mine tailings, *Imperata cylindrica*, metal fraction, phytoavailability

## Abstract

The environmental risk of potentially toxic metals in tailing soils is of universal concern. We conducted a 3-month pot experiment to research the distribution and variations of potentially toxic metals (PTMs), and the translocation and accumulation capability of these metals (Cr, Ni, Mn, Cu, Zu) in natural plants for three Fe/Mg tailing soils (serpentine-type, olivine-type and magnetite-type) with growth of a grass plant-*Imperata cylindrica*. We used comparative analysis, regression analysis and correlation analysis to process relevant experimental data. Results showed the rhizosphere tailing soils decreased from 3.70% to 16.8%, compared to the bulk soils, after growth of *Imperata cylindrica*, and the acid soluble fraction of Mn, Cu and Zn increased significantly. Cu and Zn were more bioavailable than other PTMs, especially for serpentine-type tailing soils. Linear regression analysis indicated that non-residual fractions showed different effects on metal concentrations of *Imperata cylindrica*. The non-residual metal fractions of serpentine-type and olivine-type tailing soils showed better correlations with metal concentrations in grass plants than those of magnetite-type tailing soils. We found that the chemical compositions of tailing soils showed remarkable effects on Ni and Mn compared with other elements, especially Mg and Al. Overall, the grass plant can alter the metal distribution, enhance metal bioavailability and promote land use of Fe/Mg tailing soils.

## 1. Introduction

Metal mining generates resources for industrial production and livelihood but generates substantial tailings enriched in potentially toxic metals (PTMs). In China, there are many mines that provide mineral resources for domestic and global consumers, and the pollution risk of PTMs from mines is universal [1]. These mineral resources contain various metals such as manganese (Mn), magnesium (Mg), copper (Cu), nickel (Ni), lead (Pb), chromium (Cr) and zinc (Zn). During the mining and smelting processes, these mines have generated a large number of abandoned tailings, bringing the serious environmental risk of metal pollution [2,3]. These tailings sites have attracted great attention because they occupy precious land and influence soil quality in China. For example, agricultural soils and crops were reported to be heavily contaminated with Cd, Pb, Cu and Zn in the vicinity of some mine areas [2,4]. Recognizing the characteristics and risks of PTMs in tailing soils is urgent. Fe/Mg-bearing mines are usually found in cover areas of basic and ultrabasic rocks, which are utilized to extract Fe and Mg for industrial use. Although they are characteristic of Fe/Mg-bearing minerals, they have diverse toxic metals in their tailings. The distribution and phytoavailablity of PTMs in Fe/Mg tailing soils has been unclear, especially for Fe/Mg tailing soils with different mineral characteristics [5].

Currently, the quality of tailing soils is still a concern for the government and the farmer. There are some ways to improve the quality of tailing soils. Among them, phytoremediation is a most popular way, due to the advantage of reducing metals in an environmentally friendly and cost-effective manner [6]. Plant growth can change the soil properties of the rhizosphere environment, activate metals and promote plant absorption. Because of transfer from soil to plant, PTMs are reduced in tailing soils. But this is significantly affected by the properties of the tailing soils and the metal species [7,8]. For example, changes in pH can lead to the release or precipitation of metals in soil, and organic matter can form complexes with metals, affecting metal migration. Clay content is also an important factor affecting soil metal migration [7]. Although the total metal concentration in soil was related to the metal in the plant, metal chemical fraction was more closely related to the metals in the plant [9,10]. Therefore, it is important to recognize the relationship between metal concentration in plants and metal fraction in Fe/Mg tailing soils.

In spite of lacking nutrients, some plants can still grow in tailing soils. *Imperata cylindrica* is a common widespread natural colonizer surrounding the tailings areas because of its shallow root elongation and dense fine roots, and it is widely distributed in Fe/Mg tailing soils [11]. However, few studies have focused on the metal distribution and phytoavailability of different Fe/Mg-type tailing soils. Previous research about heavy metals in the tailing soils have mainly concentrated on sulfide tailings, because these tailings had high toxic metals [2,5], while the Fe/Mg-type tailings were different, and the toxic elements (such as Cd, Hg and As) were not high in tailing soils. As the tailings formed by ultrabasic and basic rocks have high contents of Cr and Ni, it is of great significance to understand the distribution and phytoavailability in such tailings soils. Therefore, in our study, we aimed to: (1) investigate the distribution differences of PTMs in different Fe/Mg tailing soils; (2) compare the bioavailability of PTMs in plants for three Fe/Mg-type tailing soils with different mineral components; (3) explore the major influencing factors of soil properties on metal translocation and accumulation in the Fe/Mg tailing soil-plant system.

## 2. Materials and Methods

### 2.1. Collection and Preparation of Tailing Soils

The tailing soils were collected from the surface layer (0−20 cm) of three different Fe/Mg tailings piles, which were located in Lianyungang city of Jiangsu, Nanyang city of Henan and Panzhihua city of Sichuan. About 5 kg of tailing soil was packaged in a cloth sample bag and 10 packages were collected in one tailings pile. After being transported to the laboratory, these samples were air-dried and debris was removed. Then dry samples were screened by 2 mm mesh [9]. The screened samples were used for pot experiments.

### 2.2. Pot Experiment and Laboratory Incubation

The tailing soils from three sampling locations were mixed with organic fertilizers (leaves and wood chips) uniformly at a ratio of 5:1 (*w*:*w*). Then 3 kg of soil mixture was weighted and placed in a plastic pot (21 cm × 15 cm × 13.5 cm). *Imperata cylindrica* seeds were cultivated in the Hoagland solution initially and germinated into seedlings for 2 weeks (16/8 h for day/night period, 20 °C/18 °C for day/night temperature, 500 lux of light intensity). When the seedlings had grown three pairs of leaves, they were moved to the pots randomly (10 seedlings per pot), and given 60 mL of deionized water every day. At the end of the three-month growth period, these plants were taken out of the pot for analysis, and rhizosphere soils were simultaneously collected. Triplicates of tailing soils for each type were handled in the pot experiment. All pot experimental works were performed in the greenhouse of Hohai University in Nanjing, China.

### 2.3. Soil Physical and Chemical Analysis

The main physicochemical properties of tailing soil samples were determined by the following methods. The pH of the sample was detected using a pH meter (SX751, Shanghai Sanxin meter factory, Shanghai, China) at a soil-water ratio of 1:2.5 (*w*:*v*) [11]. Organic matter content was determined by the chromic acid titration method and soil particle-size was determined using a laser particle analyzer (Beckman Coulter Corp., Fullerton, CA, USA). Tailing soil samples were then analyzed using an X-ray fluorescence instrument (XRF, PW2440, Philips Company, Amsterdam, Netherlands) to determine the compositions of major elements (through 0.1 mesh sieve). These samples were digested with HF-HNO_3_-HClO_4_ at a ratio of 2:1:1 (*v*:*v*:*v*), and then analyzed for total concentrations of PTMs by ICP-OES (ICAP 6000 Serises, Thermo, Waltham, MA, USA). Two soil standard samples were analyzed at the same time to ensure the accuracy of the determination. The speciation of PTMs in samples was obtained by the modified BCR three-step sequential extraction procedure, and these metal fractions were determined by ICP-MS (IRIS Advantage, Thermo, Waltham, MA, USA). This modified BCR extraction procedure has been described in detail in another reference [12]. Based on this BCR method, PTMs in tailing soils can be separated into four fractions: weak acid soluble fraction (F1, exchangeable and carbonate-bound metal fraction), reducible fraction (F2, Fe-Mn oxide-bound metal fraction), oxidisable fraction (F3, sulfide and organic matter-bound metal fraction) and residual fraction (F4, lattice-binding metal fraction).

### 2.4. Plant Sample Analysis and Transfer Factor Calculation

Plant samples in pot experiments were washed sequentially by tap water, 10 mM/L EDTA and deionized water. Roots and shoots were cut with a stainless steel knife. Five grams of plant tissue was cut into small pieces and dried in an oven at 105 °C for 24 h, then ash was obtained at 500 °C in a muffle furnace [2]. 0.1 g of ash was weighed and digested with HNO_3_-H_2_O_2_ at a ratio of 4:1 (*v*:*v*) to determine the concentrations of Cr, Mn, Ni, Cu and Zn by ICP-MS. Metal concentrations in the plant samples were expressed as dry weight (mg/kg).

The translocation factor (TF) and bioaccumulation factor (BF) of PTMs from soil to plant were calculated as follows:

TF = metal concentration in root/metal concentration in soil(1)

BF = metal concentration in shoot/metal concentration in soil(2)

### 2.5. Statistical Analysis

Statistical analysis was done on Microsoft Office Excel 2010 (Microsoft, Seattle, WA, USA). Differences of tailing soil properties and metal concentrations in the samples were examined by the independent-variable test. Correlations between metal concentrations and soil properties were assessed by the Spearman method. Multiple stepwise linear regression analysis was carried out with SPSS 21.0 (IBM Inc., Chicago, IL, USA). The figures were drawn using SigmaPlot10.0 (Jandel Corporation, San Jose, CA, USA).

## 3. Results and Discussion

### 3.1. Physicochemical Property and Heavy Metal Concentration of Tailing Soils

The physicochemical properties of three typical tailing soils are presented in Table 1. The pH values of three tailing soils showed weak-alkaline characteristics, ranging from 7.38 to 7.65. Higher concentrations of Fe and Mg in the samples indicated that these samples showed the characteristics of mafic tailing soils. Although these tailings come from ultrabasic and basic rocks dominated by dark minerals, the mineral compositions of different tailings are different due to differences in mineral formation in varying temperature, pressure and water content. Serpentine-type tailing soils (STS) and olivine-type tailing soils (OTS) showed abundant Mg, while magnetite-type tailing soils (MTS) showed abundant Fe. In accordance with the pH characteristics, the highest concentrations of Ca and Mg were found in OTS. This was due to the abundant content of calcareous minerals (calcite and dolomite) in the sample [5]. The average values of organic matter were 2.08% for STS, 1.18% for OTS and 0.88% for MTS. Significant differences were found for these tailing soils (Table 1). Rodríguez et al. proposed that the covering level of vegetation was responsible for the changes in OM content in soils [5]. MTS samples showed higher Al, Fe and Ca content and lower Mg content than STS and OTS samples, which reflected the differences in mineral components. The particle-size distribution of tailing soils showed a little difference, which wasn’t associated with the chemical compositions. This feature should come from the similar smelting process.

**Figure 1:**
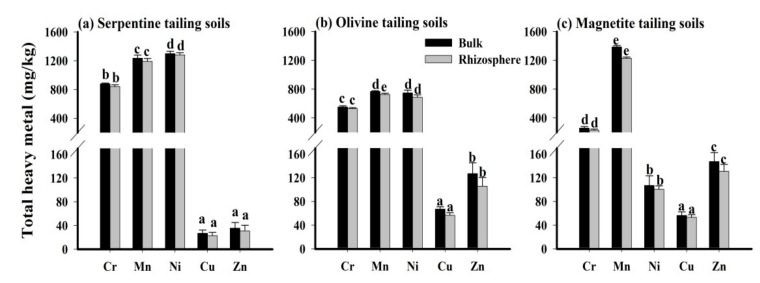
Total heavy metal concentrations in tailing soils. (**a**) Serpentine tailing soils, (**b**) Olivine tailing soils and (**c**) Magnetite tailing soils after growth of *Imperata Cylindrica*. Bulk: Bulk tailing soils; Rhizosphere: Rhizosphere tailing soils. The letters in the figure represent concentration differences among different heavy metals (*p* < 0.05). The same letter represents no significant difference.

The distributions of heavy metal concentrations in tailing soils are shown in Figure 1. As mentioned above, the concentrations of Cd and Hg in Fe/Mg tailing soils are very low, and the metal geochemical forms cannot be analyzed correctly. However, the studied metals (Cr, Ni, Mn, Cu, Zn) in tailing soils can obtain the metal forms better. The concentrations of Cr in bulk tailing soils were nearly 1−3 times higher than the value of the Grade II environmental quality standard of soils (300 mg/kg) in China (GB 15618-1995, National Environmental Protection Agency of China) and the concentrations of Ni were nearly 1−24 times above the Grade II environmental quality standard (50 mg/kg). But the concentrations of Cu and Zn in tailing soils were slightly higher than average values in paddy soils (Cu ≤ 35 mg/kg, Zn ≤ 100 mg/kg), and significantly lower than the standard values mentioned above (Cu = 100 mg/kg, Zn = 250 mg/kg). Obviously, the mine type significantly affected the metal concentrations of tailing soils [3]. In our studies, higher concentrations of Cr and Ni were associated with STS, while higher Cu concentrations were associated with OTS. However, relatively low concentrations of PTMs existed in MTS, except for Mn.

In order to observe the changes of PTMs in tailing soils after plant growth, the concentrations of PTMs in bulk tailing soils and rhizosphere tailing soils were detected separately. After the growth of *Imperata cylindrica*, a decrease in total metal concentrations in rhizosphere soils was observed for the studied metals compared to bulk soils. For example, the average concentrations of Cr and Ni in STS were reduced from 877 and 1298 mg/kg to 840 and 1280 mg/kg, respectively. A similar tendency was also found in another study, which observed that the concentrations of Cu, Pb, Cd and Zn in rhizosphere soils were lower than those of bulk soils near the Pb/Zn smelter [13]. But the descend ranges of PTMs in rhizosphere soils changed for three-type tailing soils. OTS showed a large range and MTS showed a relatively small range.

The significant differences in soil properties and metal concentrations in three-type tailing soils were associated with their mineral components. As observed in the STS, higher levels of Cr and Ni may generate from its parent rock (serpentinite). Hseu reported that the serpentinitic soils were enriched in lithiogenic Cr and Ni [14], and the relatively lower pH will cause the higher risk of PTMs in STS. Comparably, MTS contained a large amount of Fe as shown in Table 1, due to its predominant component of magnetite. As Fe in MTS can be replaced by Ni at a low level, and Ni is not strongly bound to Fe-oxyhydroxide surface, it easily releases into soil water. The risk will increase because organic acids enhance the bioavailable metals in the rhizosphere environment and promote the plant absorption [15]. Among PTMs, Zn in rhizosphere soils for OTS had the highest decrease of 16.8% as shown in Figure 1. This was consistent with the results of Riesen and Feller [16], who reported that Zn was rapidly transported in the rhizosphere environment compared to other PTMs. Therefore, apart from the physicochemical characteristics of tailing soils, the metal intrinsic property also significantly affects its distribution in rhizosphere soil.

### 3.2. Geochemical Fractions of Potentially Toxic Metals in Bulk and Rhizosphere Tailing Soils

**Figure 2:**
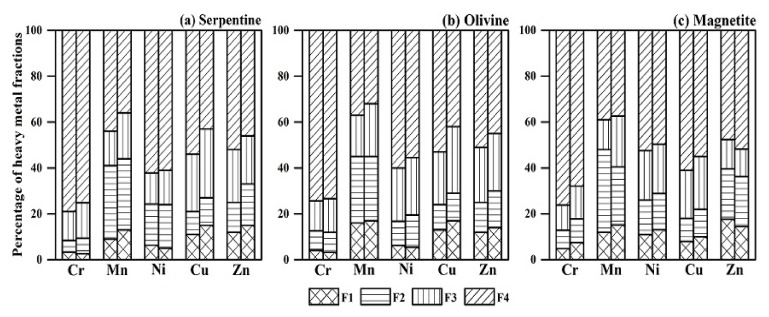
Speciation distributions of heavy metals in different tailing soils. (**a**) Serpentine, (**b**) Olivine and (**c**) Magnetite tailing soils before and after growth of *Imperata Cylindrica*. The vertical bars with patterns for each metal in the left and the right represent samples of bulk and rhizosphere tailing soils. The meanings of F1, F2, F3 and F4 are shown in Section 2.3.

Though total concentrations of PTMs can be used as an indicator of soil pollution, many studies propose that the geochemical forms of PTMs in soils are more effective for assessing the ecological risk [17,18]. The available concentrations of PTMs in tailing soils will generate the different plant absorption and toxic risks [19]. Therefore, we will investigate the metal speciation in bulk and rhizosphere tailing soils to illustrate the changes of metal fractions after plant growth. These results are shown in Figure 2.

F1 fraction was considered to be the labile form and bioavailable fraction for metal- contaminated soils, it was also easy influenced by soil properties [20]. The percentage of F1 fraction was generally lower than 20%, due to the more stabilized metals in tailing soils. Among the studied metals, Cr and Ni accounted for the lower percentages of F1 fraction compared to other metals (Mn, Cu, Zn). It was obvious that the proportions of F1 fraction was affected by the tailings type. For example, the largest F1 fractions of Cr and Ni occupied 4.8% and 6.5%, respectively, in MTS. While the largest F1 fractions of Mn, Cu and Zn occupied 13%, 16% and 12%, respectively, in OTS (Figure 2). The residual fractions (F4) of PTMs were always dominant in tailing soils. This fact has been reported by some authors in the study of mine soils [5,20]. And the F4 percentages of Cr and Ni were higher than those of other metals (Figure 2). The other chemical forms (F2, F3 fraction) varied significantly among different metals and tailing types. Results showed the largest proportions of the F2 and F3 fractions exhibited in STS for Cu and Zn, in OTS for Mn and in MTS for Cr and Ni. It is indicated that the distributions of metal fractions in tailing soils are dependent on the metal nature and matrix properties [21]. 

As samples were collected from the mafic mines with abundant basic minerals, these minerals contained Cr and Ni in crystal form. For example, the higher proportions of F4 fraction for Ni and Cr existed in MTS, and were associated with the V-Ti magnetite. But Mn, Cu and Zn do not generally exist in mineral crystal, and are mainly held through the electrostatic attraction on the mineral surface of clay minerals or organic matter [5,22]. For Cu and Zn, the adsorption capacity was significantly controlled by their oxidation status, which was generally driven by divalent iron [23]. Thus, comparing with other metals in tailing soils, Cu and Zn in tailing soils showed higher proportions of F1 fraction, especially for STS and OTS.

After the growth of *Imperata cylindrica*, heavy metal fractions varied between bulk and rhizosphere tailing soils. For Mn, Cu and Zn in three-type tailing soils, the percentages of F1 fraction increased in rhizosphere soils, compared to their fractions in bulk soils. For Cu and Mn, the percentages of F1 fraction significantly increased from 11.2% to 15.3% and from 9.2% to 12.5%, respectively, in STS. But slight declines of exchangeable Cr and Ni in rhizosphere soil were observed in STS and OTS. Interestingly, the percentages of F4 fraction decreased in rhizosphere soils for all metals. For example, the percentages of residual Cr in rhizosphere MTS and Cu in rhizosphere OTS reduced from 78% to 71.2% and from 58.7% to 45.6%, respectively, compared to the bulk soil (Figure 2). According to Figure 2, changes in reducible and oxidizable metal fractions (F2 and F3) don’t show explicit trends like F1 fraction. As opposed to bulk soils, metal fractions in rhizosphere soils were influenced by the low-molecular-weight organic acids (acetic, lactic, malic acids) from plant roots and microbe excretion [24,25]. Previous studies indicated more extractable metals would be produced in rhizosphere soils due to metal−organic complexes [13]. Obvious results were also observed for Zn and Mn in STS, Cu in OTS, and Ni and Cr in MTS in our study (Figure 2). Thus, the mobilization behavior of PTMs in tailing soils depends not only on the metal species, but also on the tailing-type soils [26].

### 3.3. Correlations Between Soil Metal Fraction and Plant Metal Concentration

**Figure 3:**
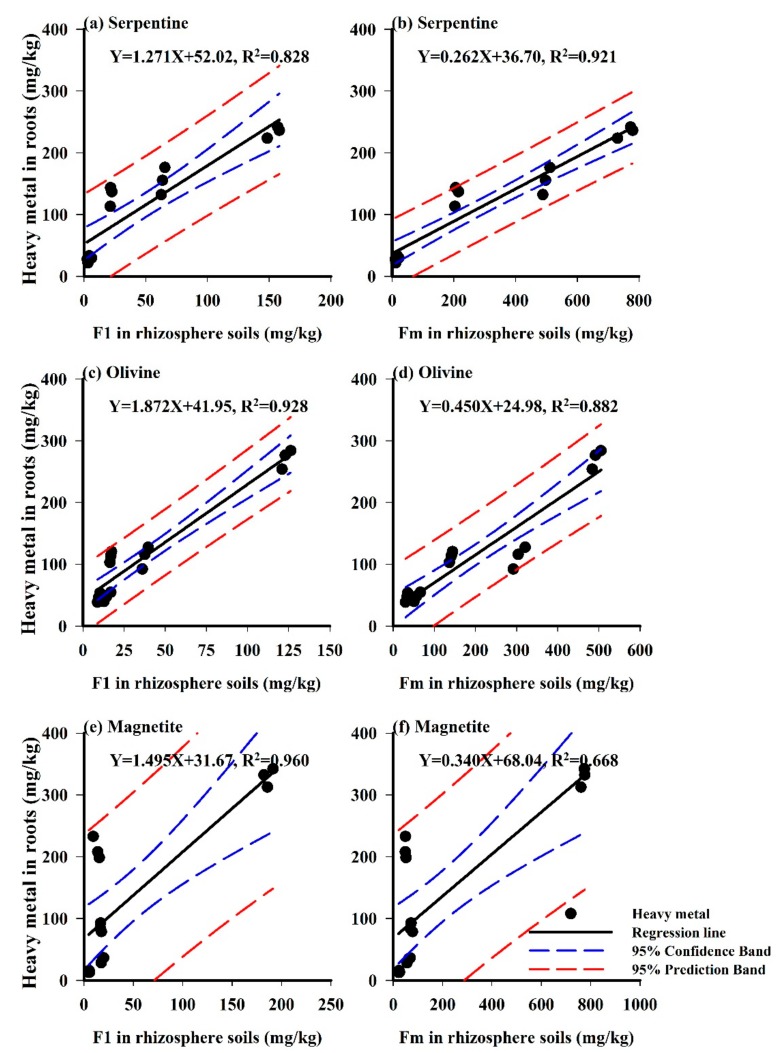
Diagrams of linear regression analysis between heavy metals in roots and metal fractions in three-type tailing soils. The letters (**a**–**f**) indicate the order of the graph for illustration in the text.

**Figure 4:**
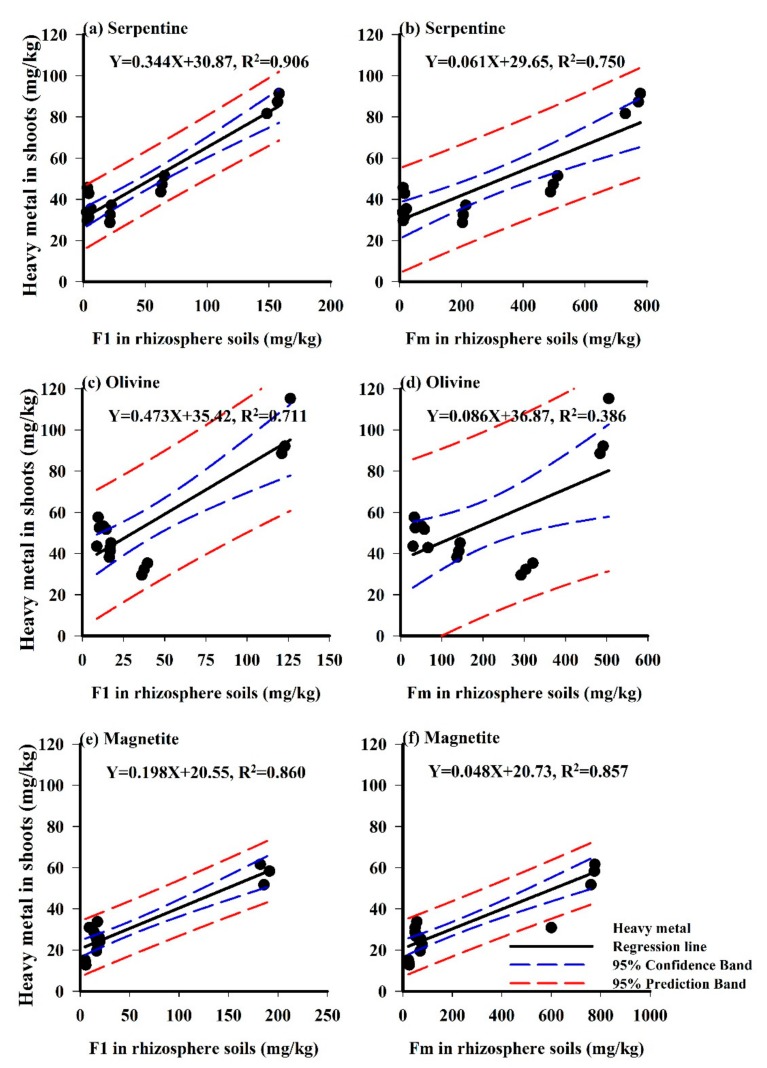
Diagrams of linear regression analysis between heavy metals in shoots and metal fractions in three-type tailing soils. The letters (**a**–**f**) indicate the order of the graph for illustration in the text.

F1 fraction of soil metal is considered as the labile and absorbable component for plants. But part metals of F2 and F3 fractions are also absorbable in some circumstances. It is important to recognise the relationships between the metal fraction in soils and the metal concentration in plants for remediation objectives [5]. Here we observed the correlations between metal F1 and Fm (F1+F2+F3) fractions (non-residual fraction) and metal concentrations in *Imperata cylindrica* for different tailing soils in order to realize the discrepant absorption capacity of vegetation. In previous studies, the metal fractions of rhizosphere soils were demonstrated to be important for the evaluation of heavy metal bioavailability, especially for predicting metal accumulation in plants [27]. Therefore, we adopted a regression analysis of heavy metal concentrations in tailing soils, roots and shoots for metal translocation and accumulation analysis in the soil−plant system, which are shown in Figure 3 and Figure 4. Results showed that significant differences existed in the metal translocation and accumulation of *Imperata cylindrica* in different tailing-type soils. Figure 3 and Figure 4 illustrate the correlations between the heavy metals of F1, Fm fractions and the heavy metals in plant roots and shoots. The points near the regression line indicate that heavy metal forms had a significant influence on heavy metals in plants, while the scattered points near the regression line indicate a relatively weak influence.

Consistent with previous studies [28,29], roots had higher metal concentrations than shoots in plants. In our research, roots were washed with EDTA, which had removed the metals associated with the root cell wall (apoplastic metals). Thus, the root metal accumulation exhibited metal absorption rather than sorption [30]. Results indicated that metal concentrations in roots were closely related with F1 and Fm fractions of tailing soils, especially for STS and OTS. However, for STS, the heavy metals in plants were more correlated with Fm, which indicated that sometimes the non-residue fraction can better explain the bioavailability of metals in tailing soils.

Previous studies demonstrated that the metal concentrations in shoots were obviously lower than those in roots, and they were not good indicators for metal bioavailability in soils [31]. This is because most PTMs can be kept in roots and detoxified through complexing with various ligands such as organic acids or metal-binding peptides, thus hindering heavy metal transport to the aerial portion of plants [32]. But in Table 2, we noticed the concentrations of Cu and Zn in shoots were higher than those in roots, maybe because they are essential elements for plant growth. Meanwhile, we noticed that differences in metal concentrations in roots varied among different tailing soils. For example, the highest Ni concentration in roots, 254.5 mg/kg, was detected in STS, while the lowest Ni concentration in roots, 43.0 mg/kg, existed in MTS. As opposed to other metals, higher concentrations of Cu and Zn were observed in shoots than in roots, especially for STS and OTS (Table 2). These results suggested that *Imperata cylindrica* was more efficient in both Cu and Zn translocation from root to shoot [33]. 

Based on the correlation analyses in Figure 4, the metal concentrations in shoots of *Imperata cylindrica* trended to be better associated with metal fractions in MTS (Figure 4e,f), but more weakly associated with OTS (Figure 4c,d). As shown in Figure 4, the correlation coefficients displayed relatively small values compared with Figure 3, which indicates discrepant metal translocation and accumulation in plants. It was found that significantly different correlation coefficients for Figure 3 and Figure 4 existed in MTS, which indicates metals in MTS entered into the aerial part more easily than those in STS and OTS. The correlations between metal concentrations in tailing soils and shoots was not consistent with those between metal concentrations in tailing soils and roots. It is obvious that metal translocation and accumulation is affected by properties and mineral compositions of tailing soils, although diversity also exists in different metals.

### 3.4. Influence of Tailing Soils Properties on Metal Transfer Capability

Transfer capability of PTMs in the soil−plant system is an indicator for evaluating metal accumulation in plants, and also shows the risk degree of vegetation damage exposed to pollution soils [34,35]. The transfer process is influenced by various factors, such as soil properties, plant species and metal characteristics. In many studies, the translocation factor (TF) and bioaccumulation factor (BF) were used to indicate the metal transfer capability [34]. Thus, we assessed these transfer abilities (TFs and BFs) for three-type tailing soils in our study, which is shown in Table 3. These results showed that TF and BF values varied among different tailing soils. Cu and Zn had higher TF values than other metals in STS and OTS, but Ni had the highest TF values in MTS. The elements with lowest TF value were Cr, Ni and Mn for STS, Zn for OTS and Cu for MTS, which shows the differences in metal occurrence state in tailing soils.

In order to further discuss the different effects of tailing soil properties on metal transfer, we analyzed the correlations between TF and BF values and main soil properties, which are shown in Table 4. Results showed that chemical compositions had significant effects on metal TF and BF variations, followed by organic matter and pH. But the soil structure did not show significant effects on the metal translocation and accumulation of tailing soils, although the silt fraction revealed good correlations with TF-Cr and TF-Zn. These circumstances showed tailing compositions were important for metal translocation and accumulation in *Imperata cylindrica.*

According to Table 4, Al, Fe and Ca showed significant positive correlations with Ni translocation, and negative correlations with Mn translocation, but Mg showed the opposite. These situations indicate STS and OTS promoted Ni translocation and impeded Mn translocation, and MTS promoted Mn translocation. However, chemical compositions showed weak correlations with Cu, Zn and Cr, which indicates there was less effect on the translocation of these metals.

Metals in tailing soils can adsorb onto solid minerals and complex with organic substances, decreasing the metals mobility in soils [36]. However, soil organic matter, such as fulvic acids, may enhance the metal transfer capability through the formation of soluble metal−organic complexes, facilitating metal accumulation in plants and their transport to the aerial part [37], as we have observed Cu and Zn accumulation in shoots. These results indicate that the influence of soil properties on plant uptake of heavy metals is not only dependent on their occurrence status, but also their metal species [38]. For most discussed soil compositions, they can exist as cations and anions in soil solutions. These ions are helpful in plant growth, and can thus influence metal translocation and accumulation [39,40]. Compared to TF values, the BF values reveal more close correlations with soil chemical compositions, which indicates that the macro and micro-metals are simultaneously transported and absorbed by aerial parts of *Imperata cylindrica*. Similar results have been reported by some authors in research of mine soils [28,40].

## 4. Conclusions

Fe/Mg tailings are a unique type of tailing that have not previously received attention. Due to the differences in mineral composition, the PTMs in different types of tailing soils and their migration and transformation characteristics were studied. As a whole, the proportions of metal fraction change significantly for Ni, Cu and Zn, but change weakly for Cr and Mn. *Imperata cylindrica* is a common plant growing in Fe/Mg tailings areas. It shows discrepant translocation and accumulation capabilities of PTMs (Cr, Mn, Ni, Cu and Zn) in different tailing soils. Linear regression analysis showed that the differences in translocation and accumulation of PTMs are significant for STS and OTS, but indistinctive for MTS. It is indicated that the mineral components of tailing soils have an important role in metal translocation and accumulation. Moreover, the correlation analysis between TF and BF values and tailing soil properties shows F1 component is not a good indicator for the metal migration and absorption capacity in plants sometimes, but should take into account the fraction of non-residue metals (Fm), especially for tailing soils. Our results show *Imperata cylindrica* is more effective in translocation and accumulation of Ni, Cu and Zn from soil to aerial parts, especially for Ni in MTS, Cu and Zn in STS and OTS. It is feasible to handle *Imperata cylindrica* as a pioneer plant for restoration of the mafic tailing soils.

## Figures and Tables

**Table 1:** Physicochemical properties of three-type tailing soils in this study.

Type	pH	OM (%)	Particle Size Distribution (%)			Major Element (%)			
			Clay (<2 μm)	Silt (2–50 μm)	Sand (>50 μm)	Al	Fe	Ca	Mg
S	7.38 ± 0.04a	2.08 ± 0.04c	1.19 ± 0.29a	18.2 ± 1.85a	80.6 ± 2.14a	0.97 ± 0.18a	4.88 ± 0.34a	0.71 ± 1.01a	23.1 ± 3.02b
O	7.72 ± 0.10b	1.18 ± 0.06b	1.15 ± 0.22a	13.6 ± 3.06a	85.3 ± 3.27a	0.27 ± 0.04a	5.61 ± 0.78a	0.93 ± 0.40a	24.9 ± 1.18b
M	7.65 ± 0.12b	0.88 ± 0.10a	1.08 ± 0.19a	15.7 ± 3.15a	83.3 ± 3.34a	4.15 ± 0.79b	15.6 ± 3.10b	8.29 ± 1.21b	5.72 ± 0.52a

Data expression is mean value ± SD. S: Serpentine tailing soils from Jiangsu Province; O: Olivine tailing soils from Henan Province; M: Magnetite tailing soils from Sichuan Province. The letters represent differences among different tailings types (*p* < 0.05).

**Table 2:** Heavy metal concentrations in shoots and roots of *Imperata Cylindrica* grown in three-type tailing soils (mg/kg).

Type	Cr	Mn	Ni	Cu	Zn
Serpentine					
Root	131.2 ± 15.9a	233.8 ± 9.18c	254.5 ± 22.0a	30.0 ±2.77b	26.3 ± 4.01c
Shoot	32.8 ± 4.21b	86.7 ± 4.87b	140.7 ±12.4a	40.7 ± 6.22ab	32.1 ± 2.98b
Olivine					
Root	112.2 ± 8.86b	271.4 ± 15.6b	211.8 ± 18.0b	46.2 ± 7.50a	47.4 ± 7.58a
Shoot	41.5 ± 3.46a	98.6 ± 14.5a	89.0 ± 6.51b	51.2 ± 7.14a	49.2 ± 5.62a
Magnetite					
Root	85.0 ± 6.99c	328.9 ± 15.0a	43.0 ± 4.61c	13.8 ± 1.55c	33.3 ± 4.34b
Shoot	22.4 ± 2.90c	57.2 ± 5.04c	23.8 ± 2.10c	14.1 ± 1.27c	28.0 ± 5.01b

Data expression is mean value ± SD (3 samples). The letters represent differences among element concentrations in roots or shoots (*p* < 0.05).

**Table 3:** Bioaccumulation and translocation factors of heavy metals in *Imperata Cylindrica* grown in three-type tailing soils.

Type	Cr	Mn	Ni	Cu	Zn
Serpentine					
TF	0.150 ± 0.020cC	0.189 ± 0.004cC	0.196 ± 0.012cC	1.145 ± 0.130aA	0.762 ± 0.105bA
BF	0.037 ± 0.005dC	0.070 ± 0.001cB	0.108 ± 0.009cB	1.551 ± 0.264aA	0.938 ± 0.164bA
Olivine					
TF	0.202 ± 0.010cB	0.353 ± 0.016bA	0.283 ± 0.011cB	0.686 ± 0.071aB	0.373 ± 0.009bB
BF	0.075 ± 0.004dB	0.128 ± 0.017cA	0.119 ± 0.008cB	0.763 ± 0.087aB	0.397 ± 0.097bB
Magnetite					
TF	0.332 ± 0.046bA	0.237 ± 0.009cB	0.870 ± 0.203aA	0.247 ± 0.037cC	0.388 ± 0.015bB
BF	0.087 ± 0.011cA	0.041 ± 0.003cB	0.478 ± 0.099aA	0.253 ± 0.049bC	0.335 ± 0.102bB

Note: Lowercase letters represent differences among elements (*p* < 0.05), while capital letters indicate differences among tailing soils (*p* < 0.05). These comparisons are for TF and BF respectively. BFs of metals generally showed lower values compared to TFs of metals. But Cu and Zn had higher BF values than TF values, especially for STS and OTS, which indicates *Imperata cylindrica* had higher efficient accumulation of Cu and Zn in shoots. But for Cr, Mn and Ni, the BF values were less than 0.15, except Ni in MTS, indicating a lower enrichment ability of *Imperata cylindrica* for these elements. In comparison, Cu was easily accumulated in *Imperata cylindrica* for STS and OTS, Zn was easily accumulated by *Imperata cylindrica* in OTS, and Ni was easily accumulated by *Imperata cylindrica* in MTS.

**Table 4:** Correlation coefficients between bioaccumulation or translocation factors of heavy metal and physicochemical properties of tailing soils.

Parameter	TF-Cr	TF-Mn	TF-Ni	TF-Cu	TF-Zn	BF-Cr	BF-Mn	BF-Ni	BF-Cu	BF-Zn
pH	0.540	−0.342	0.270	−0.502	−0.073	0.715 *	0.314	0.310	−0.699 *	−0.668 *
OM	−0.343	0.716 *	−0.664	0.707 *	0.492	−0.956 **	0.025	−0.716 *	0.951 **	0.929 **
Clay	−0.127	0.124	−0.170	0.256	0.746 *	−0.292	−0.026	−0.250	0.304	0.502
Silt	−0.688 *	−0.037	−0.003	0.572	0.756 *	−0.499	−0.527	−0.097	0.453	0.629
Sand	0.663	0.027	0.014	−0.560	−0.767 *	0.493	0.503	0.109	−0.451	−0.631
Al	−0.460	−0.922 **	0.968 **	−0.491	−0.603	0.613	−0.781 *	0.963 **	−0.668 *	−0.584
Fe	−0.288	−0.950 **	0.974 **	−0.605	−0.557	0.792 *	−0.650	0.962 **	−0.782 *	−0.685 *
Ca	−0.342	−0.949 **	0.950 **	−0.529	−0.536	0.707 *	−0.666	0.973 **	−0.807 **	−0.724 *
Mg	0.426	0.953 **	−0.967 **	0.506	0.541	−0.675 *	0.740 *	−0.976 **	0.754 *	0.667 *

*. There was a significant correlation at 0.05 level (both-sided text); **. There was a significant correlation at 0.01 level (both-sided text).
